# A study of SARS-COV-2 outbreaks in US federal prisons: the linkage between staff, incarcerated populations, and community transmission

**DOI:** 10.1186/s12889-022-12813-w

**Published:** 2022-03-11

**Authors:** Sherry Towers, Danielle Wallace, Jason Walker, John M. Eason, Jake R. Nelson, Tony H. Grubesic

**Affiliations:** 1 Institute for Advanced Sustainability Studies, Berliner Str. 130, 14467 Potsdam, Germany; 2 School of Criminology and Criminal Justice, Center for Violence Prevention and Community Solutions, Arizona State University, 411 N. Central Ave., Room 600, Phoenix, AZ 85004 USA; 3 Department of Sociology, Sewell Social Sciences, University of Wisconsin-Madison, 1180 Observatory Dr, Madison, WI 53706 USA; 4 Geoinformatics & Policy Analytics Laboratory School of Information, University of Texas at Austin, Austin, TX 78712 USA

**Keywords:** Coronavirus, Prisons, Pandemic, SARS-COV-2, Disease intervention strategies, Decarceration, Inmates, Community

## Abstract

**Background:**

Since the novel coronavirus SARS-COV-2 was first identified to be circulating in the US on January 20, 2020, some of the worst outbreaks have occurred within state and federal prisons. The vulnerability of incarcerated populations, and the additional threats posed to the health of prison staff and the people they contact in surrounding communities underline the need to better understand the dynamics of transmission in the inter-linked incarcerated population/staff/community sub-populations to better inform optimal control of SARS-COV-2.

**Methods:**

We examined SARS-CoV-2 case data from 101 non-administrative federal prisons between 5/18/2020 to 01/31/2021 and examined the per capita size of outbreaks in staff and the incarcerated population compared to outbreaks in the communities in the counties surrounding the prisons during the summer and winter waves of the SARS-COV-2 pandemic. We also examined the impact of decarceration on per capita rates in the staff/incarcerated/community populations.

**Results:**

For both the summer and winter waves we found significant inter-correlations between per capita rates in the outbreaks among the incarcerated population, staff, and the community.

Over-all during the pandemic, per capita rates were significantly higher in the incarcerated population than in both the staff and community (paired Student’s t-test *p* = 0.03 and *p* < 0.001, respectively). Average per capita rates of incarcerated population outbreaks were significantly associated with prison security level, ranked from lowest per capita rate to highest: High, Minimum, Medium, and Low security.

Federal prisons decreased the incarcerated population by a relative factor of 96% comparing the winter to summer wave (one SD range [90%,102%]). We found no significant impact of decarceration on per capita rates of SARS-COV-2 infection in the staff community populations, but decarceration was significantly associated with a decrease in incarcerated per capita rates during the winter wave (Negative Binomial regression *p* = 0.015).

**Conclusions:**

We found significant evidence of community/staff/incarcerated population inter-linkage of SARS-COV-2 transmission. Further study is warranted to determine which control measures aimed at the incarcerated population and/or staff are most efficacious at preventing or controlling outbreaks.

**Supplementary Information:**

The online version contains supplementary material available at 10.1186/s12889-022-12813-w.

## Background

Since the novel coronavirus SARS-COV-2 was first identified to be circulating in the US on January 20, 2020, some of the worst outbreaks have occurred within institutional settings such as homes for the elderly and in prisons and jails [1–5]. For some time, prisons have been known facilitators of the spread of infectious diseases through multiple pathways [6, 7], including the architectural structure of shared space by those incarcerated (i.e., dining areas and living spaces), mass incarceration and subsequent over-crowding, and the social interactions in the prison. For instance, previous estimates from the early 2000’s showed approximately 25% of incarcerated individuals having had a latent tuberculous infection [8–11], and between 29 to 43% of incarcerated individuals showed evidence of previous Hepatis C infections [8–11]. Large outbreaks of infectious respiratory diseases like influenza, in particular, are a constant threat [12–14].

The close quarters and environment of correctional facilities can also result in high incidence of SARS-COV-2 infections among incarcerated individuals. To date, over 1 in 4 incarcerated people in the US have tested positive for SARS-COV-2 and over 2,400 have died [15]. Controlling SARS-COV-2 infections in prisons is a critical part of “flattening the curve” [16], and outbreak and infection mitigation strategies used by prisons have largely been aimed at the incarcerated population. However, a strict focus on incarcerated individuals as carriers and transmitters of infectious diseases, including SARS-COV-2, means that containment efforts largely focused on that population ignore the potential influence of correctional staff on infections both within and outside of prisons. Prisons are not closed systems, and while the incarcerated individuals have little contact with the outside world, correctional staff members move in and out of the prison to the community daily. As such, prison staff can not only potentially bring SARS-COV-2 into the prison and facilitate spread in that environment as they move about during their workday, but also spread SARS-COV-2 to their local communities [16].

In an attempt to control the spread of SARS-COV-2 in incarcerated populations, in May, 2020 the Federal Bureau of Prisons (BoP) instituted a halt on prison visitation. In addition, federal prisons and some state prisons used decarceration in an effort to reduce crowding in the prison populations [3, 17, 18]. Even despite these attempted control measures, the per capita rate of SARS-COV-2 in prison populations in the US was three to six times higher than the average rate in the main population [1, 3]. However, to the authors’ knowledge no study to date has compared rates of SARS-COV-2 in prisons to those in the communities that directly surround them, and the effects that control measures such as decarceration might have on community transmission of SARS-COV-2.

Here we examined the number of SARS-COV-2 cases in the incarcerated population and staff in 101 non-administrative federal prisons in the US, beginning May 18, 2020 (the date of the last BoP SARS-COV-2 directive in the spring) to Jan 31, 2021. We also examined the number of SARS-COV-2 cases in the counties surrounding the prisons. We compared the per capita rates of the outbreaks in the three sub-populations, over-all during the pandemic and separately during the summer and winter waves. We examined how incarcerated population sizes and prison security level (such as Minimum, Low, Medium and Maximum security facilities) may have affected transmission rates, and also the effect of decarceration.

In the following sections we describe the sources of data and the statistical methods used, followed by a presentation of results and discussion.

## Methods and materials

### Data

Data on SARS-COV-2 incidence among incarcerated populations and staff in 121 federal prisons between 04/16/2020 (the date of the first recorded recovery in incarcerated individuals or staff) and 1/31/2021 were obtained from the US Federal Bureau of Prisons website [19]. We excluded 18 “administrative” facilities from consideration in the analysis, because several such facilities were medical centers (and thus specifically may have SARS-COV-2 positive individuals sent there, along with individuals with health conditions that might pre-dispose them to SARS-COV-2 infection) and others were transfer facilities. Privately run facilities in the federal system were also excluded because SARS-COV-2 incidence among staff in private facilities were not reported. Incarcerated population to staff population ratios and incarcerated population for the 103 remaining federal prisons were obtained from the Bureau of Prisons website [20]. Incarcerated population to staff ratios were not available for two prisons (FCI Marianna and USP Thompson) and thus they were excluded from further analysis, leaving 101 prisons. Unfortunately, for prisons that were part of larger multi-security-level complexes, the BoP only reported the incarcerated population to staff ratios for the entire complex, which complicates calculation of per capita rates in staff. Thus, for portions of the analysis where the staff population needed to be known to calculate per capita rates we excluded prisons in complexes from our analysis, leaving a total of 65 prisons. Data on the number of covid tests and percent positive were not available from the BoP [21].

County level data on SARS-COV-2 incidence in the general population were obtained between 01/22/2020 and 1/31/2021 from the Johns Hopkins UniversityCoronavirus Resource Center [22].

On May 8, 2020 the US Department of Justice released a memo to all federal Bureau of Prisons detention centers recommending the release to home confinement of at-risk incarcerated individuals who were at low risk of offending. A subsequent memo on May 14, 2020 describing testing protocols for new admissions, and another subsequent memo on May 18, 2020 recommended significant diminishment of movements between detention centers. To ensure uniformity of comparison of data from different prisons, we restricted the analysis to data collected on or after May 18, 2020.

Because this study used aggregated public data, it was determined not to constitute human subject research by the Arizona State University institutional review board.

It is important to note that the SARS-COV-2 data for incarcerated individuals and staff in federal prisons qualitatively differ from the SARS-COV-2 data in the county main population in that the latter are the number of confirmed cases (“incidence”) per day, whereas the former are the time series of the number of incarcerated individuals and staff who have tested positive and are still positive (“prevalence”), along with the time series count of previously infected and subsequently recovered individuals and individuals who have died. Both of these time series were tallied by the BoP at various unevenly spaced time points, not necessarily daily. Unfortunately, data were not available on the total number of tests done per day on staff and incarcerated individuals in the BoP prison system.

Over a long enough time period, the sum of the incidence is approximately equal to the total number of recovered individuals (for example, once an outbreak ends, the two numbers are equal). Thus, in portions of the analysis where we compared outbreak size in prisons to outbreak size in the county main population over a particular time period, we summed the incidence within the county, and compared to the number of recovered or dead incarcerated individuals and staff over the same period; the incidence in the populations can be compared as long as there are not significant delays in the transmission dynamics between the populations over the time period of consideration (for example, the peak in incidence in one population lagging peaks in the others with a delay on the order of the length of the time period being considered).

While not relevant to this analysis because we do not analyze temporal trends, it should be noted that the time series of the number of newly infected individuals lags the prevalence time series by the average infectious period divided by two (the infectious period for SARS-COV-2 is between 11 to 20 days [23]).

By May 18, 2020 all counties with federal prisons had recorded at least one SARS-COV-2 case, with the exception of Lassen County, CA where Herlong federal prison is located (the first case was recorded in that county on May 28, 2020). Over half of federal prisons had no recorded cases in incarcerated individuals or staff before that date. Between May 18, 2020 to Jan 31, 2021 just seven prisons accounted for over 50% of all cases in incarcerated individuals and staff.

### Statistical methods

#### Per capita rates

A commonly used statistic to assess relative outbreak size and temporal dynamics is the per capita number of cases (or deaths) in a population over some time period [24, 25]. While often used, it must be cautioned that the detected number of cases in a population generally does not reflect the total number that actually occurred, particularly for diseases with a high asymptomatic rate and when testing availability is limited [24]. In addition, comparing per capita rates across different locales is complicated by the fact that-testing availability and testing protocols may be quite different in the locales. “Different locales” can not only include different prisons [21], but also different states in which they (and their surrounding communities) reside.

The SARS-COV-2 pandemic has shown multiple peaks in incidence during the time frame of this analysis. In particular, during the first calendar year there was a peak in cases in summer 2020, and another peak at the end of 2020/beginning of 2021. To ensure approximately constant transmission rates over the periods being considered, we thus divided the average peak time analysis into summer and winter waves, separated at the end of September, 2020.

#### Correlations and pair-wise comparisons

We examined the Spearman rho correlation [26] between the per capita number of cases in federal non-administrative prison incarcerated and staff populations, and to the per capita number of cases in the counties surrounding the prisons during the summer and winter waves.

We performed pairwise Student t-test [26] comparisons of the three pairwise combinations of per capita rates in staff, incarcerated individuals and the community, over-all, and during the summer and winter waves. While we examined per capita rates in this analysis in a manner similar to many other studies of SARS-COV-2 transmission in different settings, some caution in interpretation of results is warranted because of potential differing community surveillance coverage, control mandates, and community transmission environments (such as rural vs urban, for example) in the different locales [27].

#### Relationship between per capita rates to prison security level

We used population standardized Negative Binomial linear regression methods [28, 29] to assess the potential relationship of prison per capita rates of SARS-COV-2 infection in incarcerated individuals and staff to prison security level for both the summer and winter waves and also over-all during the pandemic (we describe population standardized Negative Binomial regression methodology in detail in Additional file 1: Appendix A). In the regression, we additionally controlled for community per capita rates and state as a factor level, since there is the potential that community transmission may indirectly impact transmission in the prison systems, and states may have had differing mandates for SARS-COV-2 community and workplace control measures. Using the Akaike Information Criterion (AIC) we obtained the most parsimonious explanatory model [30].

#### Assessing the effect of decarceration in reducing outbreak size

We used population standardized Negative Binomial regression to examine the potential relationship of SARS-COV-2 per capita rates in the staff, incarcerated, and community populations to the relative decarceration over the time period of interest, while controlling for a state-level factor, and prison security level as an additional factor (and also, in the case of the staff and incarcerated populations, controlling for the community per capita rates). We did this for the entire time period between 05/18/2020 to 01/31/2021, and also separately for the summer and winter wave data.

## Results

**Fig. 1:**
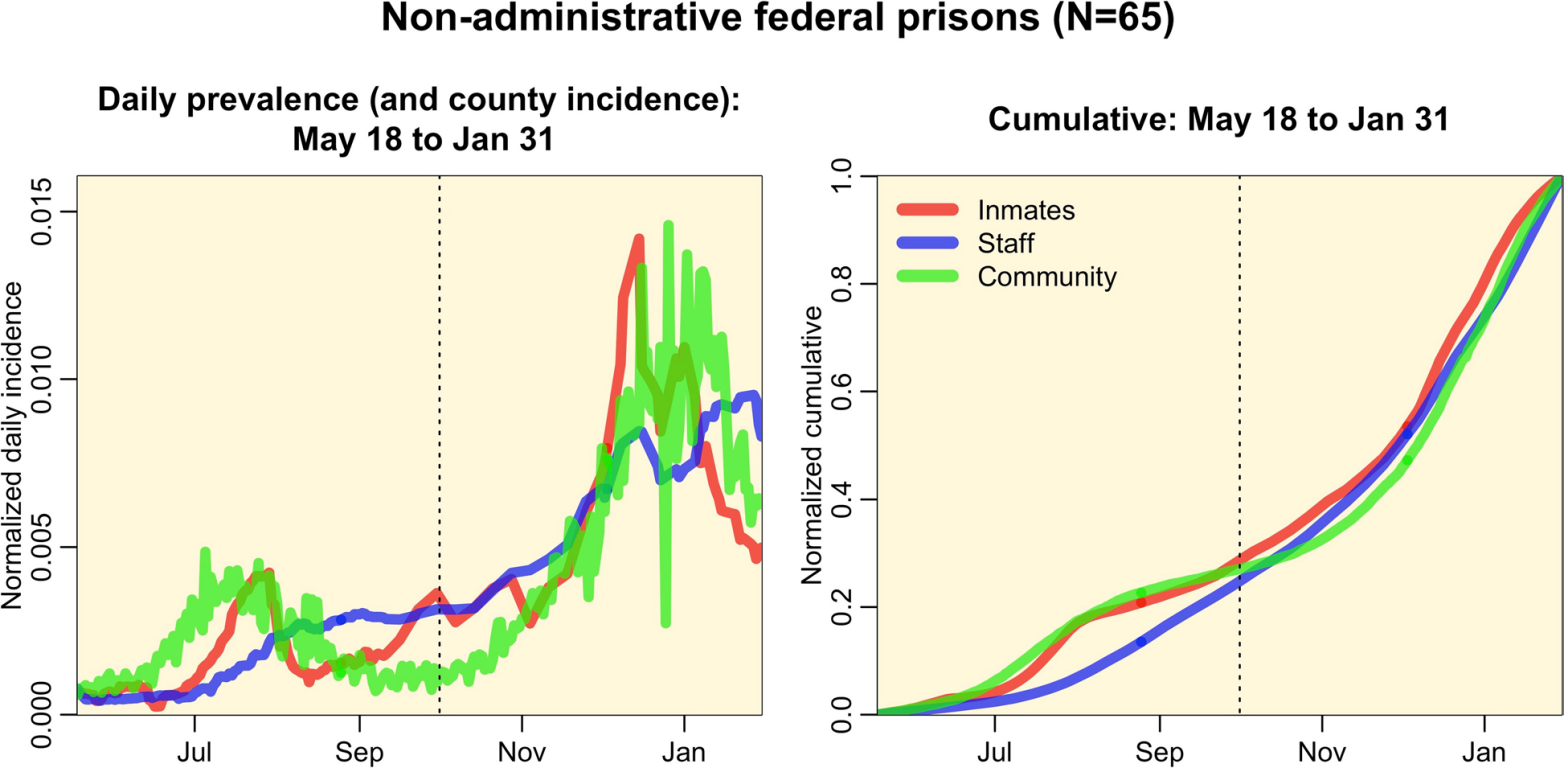
Per capita prevalence of SARS-COV-2 infections in incarcerated prison population and staff summed over non-administrative federal prisons for which the staff population is known, along with the per capita SARS-COV-2 incidence summed over the surrounding counties. For ease of comparison of the temporal patterns, the area under the curves are normalized to sum to one (however, in reality the staff and incarcerated per capita rates are around 4 times the community per capita rates, as shown in Table 1). The vertical black dotted line represents the nominal cut-off between the two waves at the end of September, 2020. Note that temporal patterns in incarcerated individuals and staff per capita rates are dominated by just a few prisons; seven prisons account for over 50% of cases in both staff and incarcerated individuals

In Fig. 1 we show the daily interpolated per capita prevalence rates of incarcerated population, staff SARS-COV-2 infections summed over federal prisons, along with the per capita incidence rates in the counties surrounding the prisons. While there were some apparent lags in peaks in incidence between the three populations [31], the lags were much smaller than the time periods under consideration over which the incidence was summed.

### Correlations between per capita rates

**Fig. 2:**
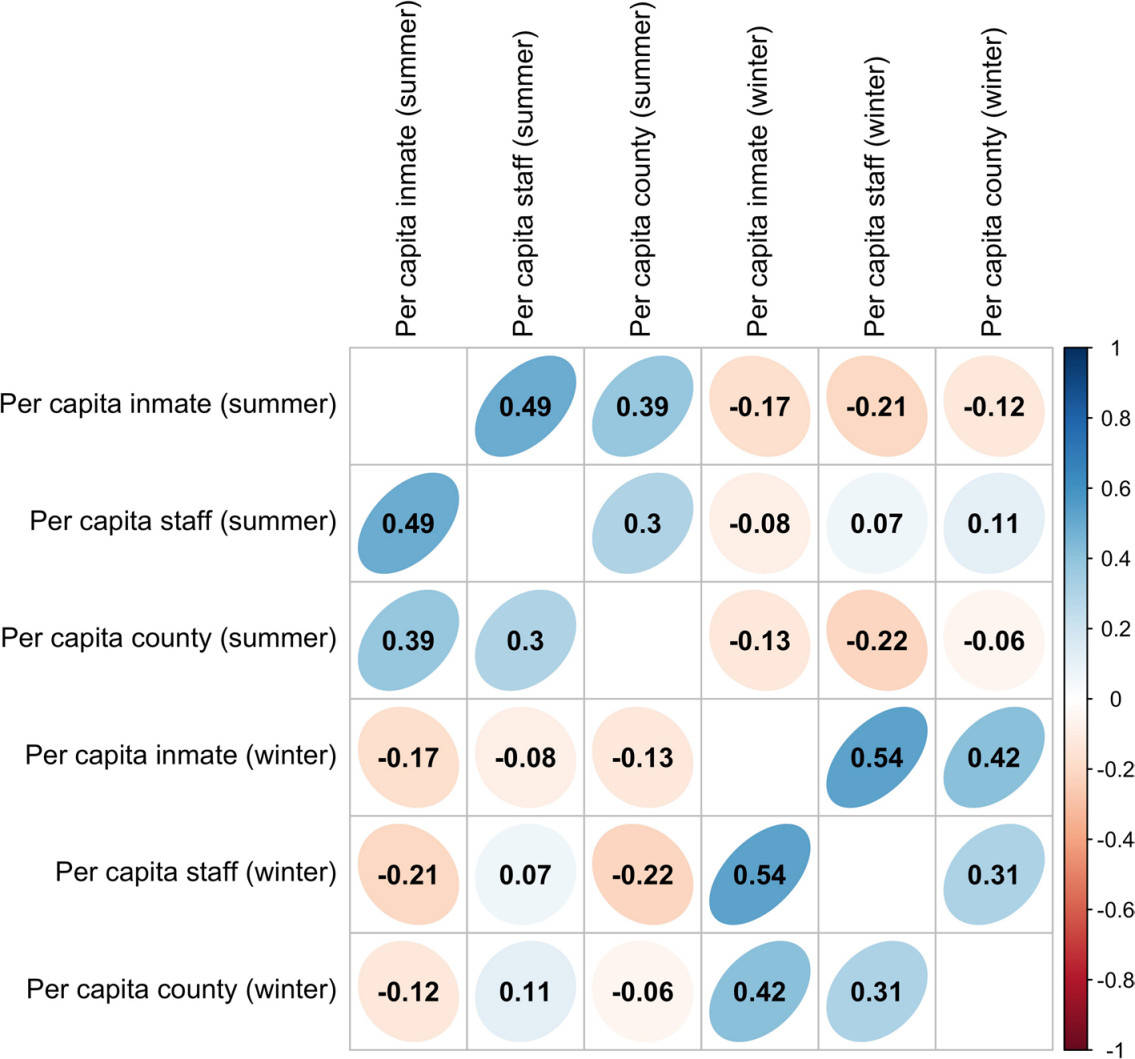
Correlogram matrix showing the correlations between per capita rates of SARS-COV-2 cases in incarcerated individuals, staff in federal non-administrative prisons, and in the surrounding counties during the summer and winter waves of the pandemic. Absolute correlations larger than 0.20 are significant to *p* < 0.05 (two-sided t-test). The more diagonal and more intensely colored the ellipsoid, the larger the absolute correlation (as shown in the scale to the right), with blue upward slanted ellipsoids representing positive correlatiosn, and red downward slanted ellipsoids representing negative correlations

In Fig. 2 we show the correlogram showing the inter-correlation between the per capita rates in incarcerated individuals and prison staff in the 101 non-administrative federal prisons, and in the population in the counties surrounding the prisons, during the summer and winter waves of the pandemic. During both waves significant positive inter-correlations are seen between the per capita rates in the sub-populations.

### Pairwise comparison of per capita rates in outbreaks in staff, incarcerated individuals, and the community

All counties surrounding federal prisons had at least one SARS-COV-2 case during the first and second waves. In contrast, 8 non-administrative federal prisons had zero cases in incarcerated individuals and 16 had zero cases in staff during the first wave. During the second wave, 0 and 7 prisons had zero cases among the incarcerated population and staff, respectively. During the first wave, 3 prisons accounted for over 50% of the total number of cases in incarcerated individuals, and 9 prisons accounted for over 50% of the total number of cases in staff. During the second wave 13 prisons accounted for over 50% of cases in incarcerated individuals, and 15 accounted for over 50% of cases in staff.

We show the pairwise comparison of the per capita rates in the incarcerated, staff and county populations in Table 1. In general, per capita rates appeared to be highest in the incarcerated populations.

**Table 1:** Comparison of the per capita rates in incarcerated individuals, staff, and county populations in non-administrative federal prisons between May 18, 2020 to Jan 31, 2021 ("Overall"), and May 18 to Sep 30, 2020 ("Summer wave"), and Oct 1, 2020 to Jan 31, 2021 ("Winter wave")

	Mean difference	Paired Student’s t-test *p*-value	Risk Ratio
Overall			
*p*^*inmate*^* – p*^*staff*^	[0.01,0.14]	0.025	1.22
*p*^*inmate*^* – p*^*county*^	[0.24,0.38]	< 0.001	4.47
*p*^*staff*^* – p*^*county*^	[0.18,0.29]	< 0.001	3.89
Summer wave			
*p*^*inmate*^* – p*^*staff*^	[-0.02,0.04]	0.53	1.15
*p*^*inmate*^* – p*^*county*^	[0.02,0.09]	< 0.001	4.32
*p*^*staff*^* – p*^*county*^	[0.03,0.06]	< 0.001	3.36
Winter wave			
*p*^*inmate*^* – p*^*staff*^	[0.01,0.12]	0.030	1.24
*p*^*inmate*^* – p*^*county*^	[0.19,0.32]	< 0.001	4.51
*p*^*staff*^* – p*^*county*^	[0.14,0.25]	< 0.001	4.03

### Relationship of per capita rates to prison security level

**Fig. 3:**
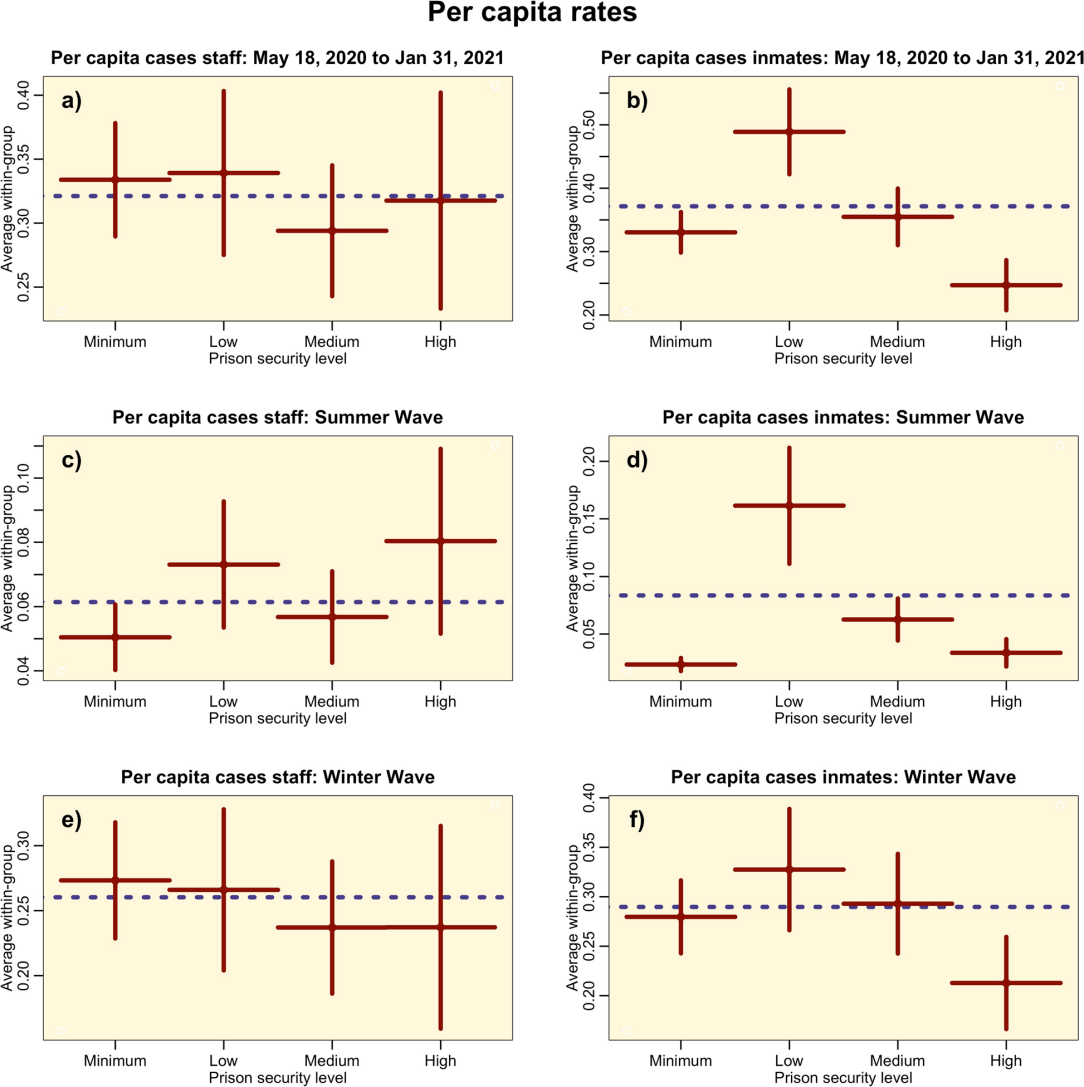
Distribution of mean SARS-COV-2 per capita rates in incarcerated and staff populations by security level for non-administrative federal prisons, over-all between May 18, 2020 to Jan 31, 2021 (top row) and for the summer and winter waves (second and third row). The vertical bars represent the standard error on the means. The dotted horizontal line represents the average over all security levels. Significant relationship to security level are seen in b) and d) (population standardized Negative Binomial factor regression *p* < 0.05 in both cases)

In Fig. 3 we show the per capita rates in staff and incarcerated populations, averaged within prison security level for the non-administrative federal prisons.

To correct for potential geographic heterogeneities, we used Negative Binomial linear regression to examine the relationship between the per capita rates controlling for the log of the per capita community rates and a state-level factor, and then chose the most parsimonious explanatory model based on the AIC. In the most parsimonious models, staff per capita rates were significantly related only to community per capita rates during the summer wave and overall (*p* < 0.001 in both cases), and only related to the state-level factor during the winter wave (*p* < 0.001). The per capita rates in the incarcerated population were significantly related only to the community per capita rates overall and during the winter wave (*p* < 0.001 in both cases), and significantly related only to prison security level and community per capita rates during the summer wave (*p* < 0.001). The results of the latter linear regression are shown in Table 2.

**Table 2:** Results of Negative Binomial linear regression of the log of the per capita rates of SARS-COV-2 cases in the incarcerated population during the summer wave on the log of the per capita rates in the surrounding community, and a factor level for the prison security level. Numbers in brackets indicate the one standard deviation uncertainty on the estimate, and entries marked with one/two/three asterisks are significant to *p* < 0.05/0.01/0.001, respectively

	Estimate
Log(community per capita)	+ 1.221(0.179)***
Factor: Minimum security	-2.264(1.049)
Factor: Low security	+ 2.921(0.758)***
Factor: Medium security	+ 2.037(0.785)**
Factor: High security	+ 1.582(0.856)*

### Effect of decarceration on per capita rates in incarcerated individuals, staff, and community

When we compared the average incarcerated populations in non-administrative federal prisons during the winter wave compared to the summer wave, we found only modest levels of decarceration; on average incarcerated populations during the winter wave were 96% of those during the summer wave (one SD range [90%,102%]).

We extended the Negative Binomial regression model of the previous section to regress the log of the staff and incarcerated per capita rates not only on the log of the per capita community rates and a state-level factor, but also the log of the relative change in the incarcerated population over the time-period of consideration. We then chose the most parsimonious explanatory model based on the AIC. The results matched those of the previous section (i.e. indicating no significant relationship to incarcerated population change), except for the incarcerated per capita rates during the winter wave, which was significantly positively related to both the log of the per capita community rates (*p* < 0.001) and the relative change in incarcerated population (*p* = 0.015; if the population went up, per capita rates increased).

Using Negative Binomial linear regression we also regressed the community per capita rates on the log of the relative change in the incarcerated population, the log of the population density, and a state-level factor, and used AIC to select the most parsimonious model. Overall, and for the summer and winter waves the final model included only the log of the population density and the state-level factor (i.e. there was no significant relationship to incarcerated population change).

## Discussion

We examined the transmission of SARS-COV-2 in the incarcerated population and staff in non-administrative federal prisons from May 18, 2020 until January 31, 2021, and compared the patterns of transmission to those observed in the communities in the counties surrounding the prisons during the summer and winter waves of the pandemic. We found significant inter-correlations between per capita rates in outbreaks in the incarcerated population, staff and communities, and even incarcerated population and surrounding community per capita rates were highly correlated, despite the fact that all prison visitation had been halted over the time-frame of the data used in this study; transmission between those two sub-populations via the staff sub-population (which contacts both) is clearly a significant factor of SARS-COV-2 transmission in prisons, underlining the fact that these are inter-linked populations from the perspective of disease transmission, and a holistic approach needs to be considered for infection control in prisons and the surrounding community that takes account of the complex inter-play of dynamics between the three populations.

During the second (but not first) wave of the pandemic we found that outbreaks in the incarcerated population had higher per capita rates than those in staff by a factor of 1.24, suggesting greater disparity in the relative reproduction numbers in the two populations as the pandemic continued. It is unclear why this might be the case, although increased control measures among the community population (like mask wearing, school closures, limitations on restaurants and gyms, etc.) may have decreased the per capita rates in the staff population to a level lower than they might have been otherwise, but meanwhile the incarcerated people were living in much the same environment as they had been during the summer wave, although it should be noted that most prisons reduced populations somewhat as the pandemic progressed through decarceration of low-risk incarcerated individuals, and there is the possibility that some institutions may have been more proactive than others in encouraging mask use. These results are contrary to those of Puglisi et al. (2020), who found that per capita rates of symptomatic infection were 40% higher in staff than incarcerated individuals in an urban jail [32]. Further study is warranted to determine the potential reasons for the disparity in results.

During the summer and winter waves of the pandemic both the incarcerated population and staff had significantly higher per capita rates of infection than outbreaks in the surrounding counties by over a factor of three. Other studies (eg; [1, 3]) have examined SARS-COV-2 per capita rates in prisons compared to the main US population, but to the authors’ knowledge, this is among the first studies to specifically examine the populations in the direct vicinity of prisons. In any study comparing prison and community per capita cases, it must be cautioned that differing SARS-COV-2 testing surveillance in prisons compared to the community might skew results [1, 33]. Unfortunately, the BoP data do not include the number of SARS-COV-2 tests done per capita in staff and the incarcerated population, which could help to better understand the magnitude of the disparities in transmission compared to the community population. In addition, socio-economic demographics and other factors such as population density in the community may be confounders.

We found significant relationship between the incarcerated population per capita rates and prison security level during the summer wave of the pandemic (but not overall or during the winter wave). In general, as security levels rise there is less contact among the incarcerated population, and incarcerated population to staff ratios go down. We found that among Low, Medium, and High security prisons, per capita rates in the incarcerated population went down by security level, in broad agreement with the findings of [34]. However, low per capita rates were also seen in Minimum security prisons, despite the high incarcerated population to staff ratios. It is unclear why this is the case, but it may be because all incarcerated individuals in federal Minimum security prisons work, whereas this is not true of the higher security prisons, and greater opportunity for group close-contact social activities in non-working prisons (particularly Low security prisons) perhaps may account for the disparities in transmission. More study is needed of the daily activities of the incarcerated population in the prisons to determine where key disparities in transmission may be occurring, and how this might inform SARS-COV-2 control strategies. The fact that later in the pandemic we found no significant relationship of incarcerated per capita rates to security level may indicate that by that point in the pandemic prisons might have established better social distancing protocols and/or more consistent use of PPE that reduced differences in transmission in the different types of prison settings.

To the authors’ knowledge, ours is among the first studies to examine the impact of decarceration on rates of SARS-COV-2 transmission among not only incarcerated populations, but also prison staff and the surrounding communities. We found that decarceration did not have a significant impact on lowering rates of SARS-COV-2 infection in the incarcerated population overall or during the summer wave, but did have a significant impact during the winter wave. These results compare to those of Vest et al. (2020) who found that 15% relative decarceration in Texas state prisons was associated with significantly lower rates of SARS-COV-2 infection and death [34]. However, the relative decarceration in federal prisons overall during the first and second waves was only 4%, and thus it may simply not have been sufficient to achieve the improvements seen in Texas. We found that decarceration also was not significantly associated with changes in per capita rates of infection of staff nor per capita rates in surrounding communities. The latter result is interesting given the inter-connected nature of the incarcerated/staff/community populations[32]; the control measure aimed at reducing crowding among the incarcerated population through release of prisoners at least does not appear to have significantly negatively impacted SARS-COV-2 transmission in the surrounding community. Further study is warranted to determine if this observation also holds for communities in the vicinity of state prisons.

### Limitations

There were many issues affecting data quality in this analysis:

- Rather than publishing total number of newly identified cases per some time-period (like day, week or month), the BoP instead publishes the number of people who have tested positive (and are still positive), along with the number recovered and number dead, and only does so sporadically and not at evenly spaced time points. The mismatch between having to compare prevalence in the prison populations to incidence in the community populations was a relatively minor complication to this analysis because we examine relatively long time periods. But the incidence vs prevalence reporting differences seriously complicate any potential future analysis of short term temporal trends in the data because incidence data lag that of prevalence by half the average infectious period. In addition, the daily reporting of community data, and irregular reporting of BoP data would also seriously complicate such an analysis. In general, the BoP surveillance data do not exhibit good epidemiological and public health practices in infectious disease reporting standards [1, 33]. Greater governmental oversight and guidance on infectious disease surveillance and reporting for such institutions is warranted to make the data more useful towards informing outbreak control, including standardization of testing protocols across institutions.
- While detailed temporal information is available on the incarcerated population per prison, estimates of staff come from one time point in mid 2020. This potentially creates some unreliability of our staff per capita rate assessments, if in fact the staff population sizes significantly varied over the time frame under consideration.
- Information of race and ethnicity of incarcerated individuals in the federal prisons, including those testing positive for SARS-COV-2 are not available, but are potentially an important factor to take into account in any analysis of transmission disparities.
- The number of SARS-COV-2 cases in the incarcerated population and staff is published at the prison level, but for prisons that are part of larger multiple-security-level complexes, the BoP only publishes staffing information for the entire complex. Thus, per capita rates for staff could not be computed for those prisons, and they had to be excluded for parts of the analysis examining staff per capita rates. Should that data be made available, it would increase the sensitivity of the analysis to detecting potential relationship of staff per capita rates of infection to factors like security level.
- The BoP does not publish the number of tests performed, only the total number of positive tests (and deaths), and it is unclear what surveillance protocols were in place, and whether these differed by prison [21]. For example, it is unknown whether regular random testing of the incarcerated population or staff was performed to identify asymptomatic cases, or whether testing was solely performed on symptomatic individuals, or even just upon request.

Further study is needed to determine why just seven out of 101 non-administrative federal prisons accounted for the majority of SARS-COV-2 cases in the incarcerated population and staff, similar to the findings of a previous study of Texas prisons [34]. While our studies shed some light on the factors that might be at play, an additional analysis of testing protocols should that data be made available would be useful.

Further study is also needed to examine the primary directionality of transmission and determine whether or not peak times of outbreaks in communities tend to precede or lag peak times in incarcerated populations. High-risk sub-populations can form a “reservoir” of infection that can continue to re-infect the broader population even when strict control measures are in place [35]. The highly complex dynamics of disease spread among inter-connected populations require mathematical models to assess the relative impacts of various proposed control measures, because some control measures aimed at one sub-population can lead to unexpectedly adverse outcomes for other populations. In future work, the authors will develop a model for incarcerated/staff/community SARS-COV-2 transmission and use it to perform in silico assessment of various potential control measures, including reducing crowding through decarceration, cohorting of incarcerated populations, quarantine, isolation, and decreased transmission through social distancing and use of PPE.

## Conclusions

We examined the transmission of SARS-COV-2 in incarcerated populations and staff in non-administrative federal prisons and compared the patterns of transmission to those observed in the communities in the counties surrounding the prisons. We found significant inter-correlations between per capita rates in outbreaks in incarcerated populations, staff and communities. We also found that decarceration of incarcerated individuals during the winter wave of the pandemic was associated with significantly lower rates of SARS-COV-2 infection in incarcerated populations. These studies can help inform infectious disease response policies in prisons that help to potentially mitigate rates of infection in incarcerated populations, staff, and the community at large.

## Supplementary Information

**Additional file 1:** **Appendix A**.

## Data Availability

The SARS-COV-2 data sets analyzed during this study for prisons and community are publicly available at https://www.bop.gov/coronavirus and https://bit.ly/39AQJJv (accessed March, 2021). No administrative permissions were required to access the files.
