# Supplementary material for: A study of SARS-COV-2 outbreaks in US federal prisons: the linkage between staff, incarcerated populations, and community transmission
Source: BMC Public Health. 2022 Mar 11;22:482. doi: 10.1186/s12889-022-12813-w (PMC8916071; doi:10.1186/s12889-022-12813-w)
Supplement: Supplementary file 1 — Additional file 1: Appendix A. [file 12889_2022_12813_MOESM1_ESM.docx]

**A study of SARS-COV-2 outbreaks in US federal prisons: the linkage between staff, incarcerated populations, and community transmission**

**Appendix A**

**A.1 Probability mass distributions for over-dispersed count data**

Least squares linear regression methods are inappropriate for count data, particularly when the number of counts is low. In such cases, regression methods based on probability distributions specific to integer data, such as the Poisson distribution, are preferred (1). However, count data are often over-dispersed relative to the Poisson model, in which case a more appropriate choice is the Negative Binomial model, which, like the Poisson distribution, includes the expected number of counts, λ, as a parameter, along with another parameter, α which is a measure of the amount of over-dispersion in the data (2). The use of a Negative Binomial regression model when data are over-dispersed ensures that the estimates of the regression coefficients have accurate confidence intervals.

The Negative Binomial probability mass distribution for observing k counts when λ are expected, with over-dispersion parameter, α, is (2)

$f_{NB}\left( k | \lambda,\alpha\right)= \frac{\Gamma(k+\alpha)}{k!\Gamma(\alpha)}\left( \frac{\lambda}{\lambda+\alpha} \right)^{k}{\left( 1+\frac{\lambda}{\alpha} \right).}^{-\alpha}$ Eqn 1

When α → ∞ the distribution approaches the Poisson distribution.

**A.2 Population standardized regression**

To examine trends in per capita rates of SARS-COV-2 infection we used population standardized regression methods. The Poisson and Negative Binomial linear regression models both assume by default a logarithmic link, thus the regression takes a log-linear form in the regressors, $x_{j}$, and regression coefficients, $\beta_{j}$:

$log\lambda= \beta_{0}+\sum_{j=1}^{J} \beta_{j}x_{j}$, Eqn 2

where J is the total number of regressors, $x_{j}$ (for example, if regressing only on time, $J=1$, and $x_{1}$ is time). The best-fit parameters are the $\beta_{j}$ which maximize the likelihood of observations.

However, when analyzing the per capita number of SARS-COV-2 cases, the number of cases we detect in a particular locale, $\lambda,$ will be proportional to the number of individuals in that locale, N. To take this into account, we perform what is known as population standardization of the model (3), with

$log\lambda= logN+\beta_{0}+\sum_{j=1}^{J} \beta_{j}x_{j},$ Eqn 3

and perform the linear regression analysis with the coefficient of the log N term fixed to one.

Because Negative Binomial linear regression inherently involves a log link for the dependent variable, one should also take the log of per capita rates used as explanatory variables. Because the per capita rates may be zero for one or more cases in the explanatory variable, we instead take the log of (x+1)/N, where x is the number of cases, and N is the population size.

**References**

1. Neter J, Kutner MH, Nachtsheim CJ, Wasserman W. Applied linear statistical models. Vol. 4. Irwin Chicago; 1996.

2. Lloyd-Smith JO. Maximum likelihood estimation of the negative binomial dispersion parameter for highly overdispersed data, with applications to infectious diseases. PLoS One. 2007;2(2):e180.

3. Osgood DW. Poisson-based regression analysis of aggregate crime rates. J Quant Criminol. 2000;16(1):21–43.
